# Demographic and clinical characteristics of carbon monoxide poisoning: nationwide data between 1999 and 2012 in Taiwan

**DOI:** 10.1186/s13049-017-0416-7

**Published:** 2017-07-14

**Authors:** Chien-Cheng Huang, Chung-Han Ho, Yi-Chen Chen, Hung-Jung Lin, Chien-Chin Hsu, Jhi-Joung Wang, Shih-Bin Su, How-Ran Guo

**Affiliations:** 1 Department of Emergency Medicine, Chi-Mei Medical Center, Tainan, Taiwan; 2 Department of Environmental and Occupational Health, College of Medicine, National Cheng Kung University, 1 Daxue Road, East District, Tainan City, 701 Taiwan; 3 Bachelor Program of Senior Service, Southern Taiwan University of Science and Technology, Tainan, Taiwan; 4 Department of Geriatrics and Gerontology, Chi-Mei Medical Center, Tainan, Taiwan; 5 Department of Occupational Medicine, Chi-Mei Medical Center, Tainan, Taiwan; 6 Department of Medical Research, Chi Mei Medical Center, Tainan, Taiwan; 7 Department of Pharmacy, Chia Nan University of Pharmacy and Science, Tainan, Taiwan; 8 Department of Biotechnology, Southern Taiwan University of Science and Technology, Tainan, Taiwan; 9 Department of Emergency Medicine, Taipei Medical University, Taipei, Taiwan; 10 Department of Leisure, Recreation and Tourism Management, Southern Taiwan University of Science and Technology, Tainan, Taiwan; 11 Department of Medical Research, Chi-Mei Medical Center, Liouying, Tainan, Taiwan; 12 Department of Occupational and Environmental Medicine, National Cheng Kung University Hospital, Tainan, Taiwan

**Keywords:** Carbon monoxide poisoning, Descriptive analysis, Nationwide

## Abstract

**Background:**

Carbon monoxide poisoning (COP) is not uncommon, but nationwide epidemiological data are limited. We conducted a study in Taiwan to fill the data gaps.

**Methods:**

We used a nationwide poisoning database to study all COP patients in Taiwan diagnosed between 1999 and 2012. We conducted descriptive analyses and compared the differences between the two sexes. In addition, we assessed the trends in suicide and mortality rates from 1999 to 2012.

**Results:**

We identified 25,912 COP patients with an almost equal female and male distribution (50.6% vs. 49.4%). The mean age was 36.0 years, and most of the patients were between 20 and 50 years old. The highest incidence rate in the year occurred during winter. While female patients were more likely to have mental disorders (35.9% vs. 28.1%, *p* < 0.001), male patients were more likely to be resulted from suicide attempts (22.9% vs. 17.7%, *p* < 0.001). In both sexes, the suicidal rate increased from 1999, reached the peak in 2007, and then decreased gradually. Hyperbaric oxygen therapy was performed in 24.2% of the patients. Neurological sequelae developed in 9.1% of the patients, and chronic respiratory failure and requirement of long-term care were observed in 5.0% and 0.1% of the patients. The 1-month and 3-month mortality rates were 1.6% and 5.0%. The 3-month mortality rate did not show significant change between 1999 and 2012.

**Discussion:**

This study showed a whole picture of COP in Taiwan, which could add to the important knowledge of this disastrous problem in public health.

**Conclusion:**

Some important findings, including higher percentages of mental disorders in female patients and suicide attempt in male patients, seasonal changes, and trends in mortality and morbidity (suicide) rates, may help developing strategies for prevention and treatment of COP.

**Electronic supplementary material:**

The online version of this article (doi:10.1186/s13049-017-0416-7) contains supplementary material, which is available to authorized users.

## Background

Carbon monoxide (CO) is a toxic product of incomplete combustion of organic matter due to insufficient oxygen supply to enable complete oxidation to carbon dioxide (CO_2_). It is usually produced in domestic or industrial fuel-burning processes such as charcoal burning, water and gas heaters, cooking equipment, motor vehicles, gas-powered furnaces, and portable generators [1–3]. CO forms carboxyhemoglobin (COHb), which has an affinity for hemoglobin 250 times greater than its affinity for oxygen; therefore, even low amounts of inhaled CO can cause severe tissue hypoxia [4]. The heart and brain, both with a high metabolic rate, are most susceptible to CO, and therefore death and neurological sequelae are the most common and disastrous complications after CO poisoning (COP) [4–8]. The frequently suggested treatment is non-rebreathing mask (NRM), which provides nearly 100% oxygen and can shorten the half-life of CO from 320 min to 80 min in normal air [5]. Despite there has been a great controversy in that whether hyperbaric oxygen therapy (HBOT) adds more benefit over NRM alone [9–12], HBOT is still suggested for patients with COP, especially in severe poisoning [5, 13]. A recent nationwide study in Taiwan reported that HBOT is associated with a lower mortality in COP patients (adjusted hazard ratio [AHR]: 0.74; 95% confidence interval [CI]: 0.67–0.81), especially in the younger patients (< 20 years) and patients with acute respiratory failure [14].

In the US, COP is the second most common cause of non-medicinal poisoning death, which accounted for about 15,000 unintentional and non-fire-related emergency department visits and almost 500 deaths annually between 2001 and 2003 [2, 15]. In addition to unintentional death, COP also accounted for more than 2,000 suicidal deaths [16]. In the Europe, a total of 140,490 COP-related deaths were reported (an estimated annual death rate of 2.2/100,000) in 28 Member States of the World Health Organization European Region between 1980 and 2008 [17]. Because CO is odorless and ultimately fatal, COP by charcoal burning is a common method of suicide in Taiwan. Between 1999 and 2009, the incidence of suicide by charcoal burning increased from 0.22 to 5.4/100,000 in Taiwan, nearly 25 times [18]. Charcoal burning became the one of the most common methods of suicide in Taiwan and Hong Kong, contributing to a 20% increase in the overall suicide rate [19]. COP-related suicide and short-term and long-term complications, including mortality, neurological sequelae, chronic respiratory failure, and long-term care, are very important issues for public health. However, nationwide epidemiology data are limited. Therefore, we conducted a study of all COP patients diagnosed between 1999 and 2012 using a nationwide poisoning database (NPD) in Taiwan to fill the data gaps.

## Methods

### Data sources

The NPD is a subset extracted from the Taiwan National Health Insurance Research Database of cases fulfilling diagnostic codes of all the poisonings including COP in Taiwan between 1999 and 2013 without adding additional data [20]. Taiwan launched a single-payer National Health Insurance program on March 1, 1995, and as of 2014, 99.9% of Taiwan’s 23 millions of residents were enrolled in the program [20]. Foreigners living in Taiwan are also eligible for this program [20]. The database contains registration files and original claim data for reimbursement [20]. Large, computerized databases derived from this system by the National Health Insurance Administration (the former Bureau of National Health Insurance, BNHI), Ministry of Health and Welfare (the former Department of Health, DOH), Taiwan, and maintained by the National Health Research Institutes, Taiwan, are provided to scientists for research purposes [20]. Because of the nearly 100% coverage rate of the National Health Insurance program, NPD could represent all the COP patients in Taiwan during this study period.

### Identification of COP patients and definitions of variables

**Fig. 1:**
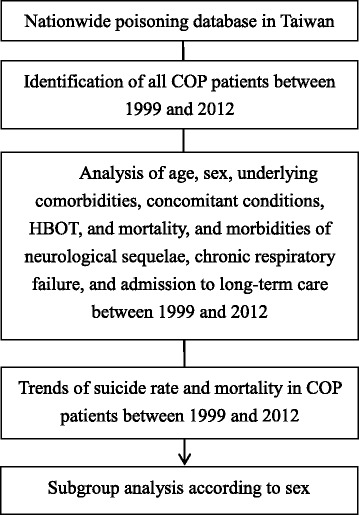
Flowchart of this study. COP, carbon monoxide poisoning; HBOT, hyperbaric oxygen therapy

From the NPD, we identified and evaluated all the new COP patients diagnosed between 1999 and 2012 (Fig. 1), which was defined as having the International Classification of Diseases, Ninth Revision, Clinical Modification (ICD-9-CM) codes of 986, E868, E952, or E982 in either hospitalization or emergency department care as one of the main diagnoses (Additional file 1: Table S1). The diagnosis of COP is based on the treating physicians under a general rule of a documented CO exposure (elevated COHb levels or ambient CO concentrations) and any of the following: headache, malaise, fatigue, forgetfulness, dizziness, loss of consciousness, confusion, visual disturbances, nausea, vomiting, cardiac ischemia, or metabolic acidosis (calculated base excess < −2.0 mmol/L, or a blood lactate level > 2.5 mmol/L). If the COHb level was <10%, the patient was eligible only if COP was the only plausible diagnosis [3–5, 9]. They were further categorized into six age groups: < 20, 20–35, 35–50, 50–65, 65–80, and >80 years. Seasons were defined as spring (March, April, and May), summer (June, July, and August), autumn (September, October, and November), and winter (December, January, and February). The underlying comorbidities studied include alcohol abuse (ICD-9-CM 291, 303, 305.0, 357.5, 425.5, 535.3, 571.0–571.3, or V113) and mental disorder (ICD-9-CM 290–319) (Additional file 1: Table S1). We divided monthly income in all the COP patients into four quartiles [21]. Because NPD has no detailed records about certain diagnoses and treatments, we used ICD-9-CM combined management codes and case classification codes as the surrogates to define the concomitant conditions such as suicide (having management codes 94.0 or 94.1, or ICD-9-CM E950-E959 in the claims for the emergency department care or hospitalization), drug poisoning, burn, acute respiratory failure, acute myocardial injury, acute hepatitis, acute renal failure, and shock (Additional file 1: Table S1). As to treatments, we defined cardiopulmonary resuscitation (CPR) by management codes 9960 or 47029C and HBOT by management codes 47054C, 9395, 59003B, 59004B, 59003A, or 59004A (Additional file 1: Table S1). For morbidities, we studied neurological sequelae, chronic respiratory failure, and long-term care (Additional file 1: Table S1). Neurological sequelae were defined as new-onset neurological or psychiatric diseases related to COP (Additional file 1: Table S1). Almost all medical expenditures covered by National Health Insurance were paid according to ICD, management, and case classification codes, and therefore using these combinations can provide the most accurate information on diagnosis and treatment in this database study. We defined four kinds of mortality according to time sequence: immediate death (the diagnosis of COP combined with out-hospital cardiac arrest ICD-9-CM 427.5, 798 or 799), short-term (1 month), intermediate-term (3 months), and long-term mortality (6 months, 1 year, 2 years, and 5 years) [6, 22, 23]. The ‘immediate death’ stands for the cases who was found dead following COP. We used ICD-9-CM in this study because the data in NPD are between 1999 and 2013, which is before the launch of ICD-10-CM in Taiwan in 2016. The details of ICD-9-CM and management codes in NPD can be found in the websites of Taiwan National Health Insurance Administration [24] and National Health Insurance Research Database [25]. All the variables and corresponding ICD-9-CM, management code or drug code, and case classification code were listed in Additional file 1: Table S1.

### Ethics statement

The Institutional Review Board at Chi-Mei Medical Center approved this study, which was also strictly conducted according to the Declaration of Helsinki. Because the NPD contains de-identified information of the patients, informed consent from the patients is waived, which does not affect the right and welfare of the patients.

### Data analysis

First, we performed a descriptive analysis of the epidemiological characteristics of the COP patients, including demographic data, season, living area, monthly income, underlying comorbidity, concomitant condition, treatment, mortality, and morbidity. Then, we made comparisons between female and male patients, using the t-test to evaluate the differences in continuous variables and the chi-square test to evaluate those in categorical variables. Furthermore, we assessed the trends of 3-month mortality rate and proportion of suicide in COP patients from 1999 to 2012 and made comparisons between female and male patients.

We used SAS 9.4 for Windows (SAS Institute, Cary, NC, USA) to perform the data analyses and conducted all statistical tests at a two-tailed significance level of 0.05.

## Results

In total, we identified 25,912 COP patients from the NPD who were diagnosed between 1999 and 2012. The proportions of female and male patients were almost equal (50.6% vs. 49.4%) (Table 1). The mean age was 36.0 years old (standard deviation = 16. 5 years), and most of the patients were in the age groups of 20–35 and 35–50 years (37.3% and 30.9%, respectively). During the year, the highest incidence of COP occurred in the winter (39.1%), followed by spring (24. 7%). According to the residential region, 61.6% patients lived in North Taiwan, followed by Center (15.9%), South (20.6%), and East Taiwan (1.9%). Most patients were in the monthly income between 1007 NTD and 21,000 NTD. The underlying comorbidity of mental disorder was 32.1%. Of the patients, 20.4% were resulted from suicide attempts, and the proportion was the highest in the spring (26.9%), followed by autumn (26.2%), winter (25.0%), and summer (21.9%). Only 4.3% of the patients had a concomitant diagnosis of burn. CPR was required in 2.1% of the patients, and HBOT was administered to 24.2% (Table 2). The proportion of patients included with a COP diagnoses solely based on the data from emergency department (ED) care was 73.3% (18,986 patients). Of the patients who was diagnosed as COP in the ED, 4,062 (21.4%) were transferred to hospitalization care, with a median length of stay (at the ED plus in the ward) of 3 days (interquartile range [IQR]: 2–6 days). The median length of stay in the patients who was diagnosed as COP during hospitalization care was also 3 days (IQR: 1–6 days). The immediate death, 1-month, 3-month, 6-month, 1-year, 2-years, and 5-years mortality rates were 0.4%, 1.6%, 5.0%, 6.0%, 7.1%, 8.6%, and 11.2%, respectively. The mortality rate during the follow-up period in the COP patients with and without HBOT was 8.8% and 14.5%, respectively [14]. The prevalence rate of neurological sequelae after COP was 9.1% in total, and the rate was 2.3% in the first 2 weeks and 6.2% at the end of the first year. In the long term, 5.0% of COP patients had chronic respiratory failure, and 0.1% required long-term care.

**Table 1:** Demographic data, underlying comorbidities, and concomitant conditions in patients of carbon monoxide poisoning in Taiwan between 1999 and 2012

Variable	Total N(%)	Female N(%)	Male N(%)	*p*-value^a^
Cases	25,912 (100.0)	13,111 (50.6)	12,801 (49.4)	
Age (years)	36.0 ± 16.5	35.2 ± 16.0	36.8 ± 16.9	<0.001
Age group (years)				< 0.001
< 20	3,462(13.4)	1,853(14.1)	1,609(12.6)	
20–35	9,657(37.3)	4,999(38.1)	4,658(36.4)	
35–50	8,015(30.9)	4,075(31.1)	3,940(30.8)	
50–65	3,291(12.7)	1,552(11.8)	1,739(13.6)	
65–80	1,081(4.2)	451(3.4)	630(4.9)	
> 80	406(1.6)	181(1.4)	225(1.8)	
Season^b^				< 0.001
Spring	6,392(24.7)	3,201(24.4)	3,191(24.9)	
Summer	4,483(17.3)	2,052(15.7)	2,431(19.0)	
Autumn	4,899(18.9)	2,296(17.5)	2,603(20.3)	
Winter	10,138(39.1)	5,562(42.4)	4,576(35.8)	
Residential region				< 0.001
North	15,954(61.6)	8,395(64.0)	7,559(59.1)	
Center	4,123(15.9)	1,959(14.9)	2,164(16.9)	
South	5,346(20.6)	2, 535(19.3)	2,811(22.0)	
East	489(1.9)	222(1.7)	267(2.1)	
Monthly income^c,d^				
< 1,007 NTD	6,635 (25.6)	3,820 (29.1)	2,815 (22.0)	< 0.001
1,007 NTD − 16,500 NTD	6,848 (26.4)	3,039 (23.2)	3,809 (29.7)	
16,500 NTD − 21,000 NTD	5,985 (23.1)	3,117(23.8)	2,869 (22.4)	
> 21,000 NTD	6,444 (24.9)	3,135 (23.9)	3,309 (25.9)	
Underlying comorbidity				
Alcohol abuse	950 (3.7)	265 (2.0)	685 (5.4)	< 0.001
Mental disorder	8,304 (32.1)	4,703 (35.9)	3,601 (28.1)	< 0.001
Concomitant condition				
Suicide	5,274 (20.4)	2,339 (17.8)	2,935 (22.9)	< 0.001
Drug poisoning	272 (1.1)	141 (1.1)	131 (1.0)	0.681
Burn	1,118 (4.3)	431 (3.3)	687 (5.4)	< 0.001
Acute respiratory failure	1,883 (7.3)	784 (6.0)	1,099 (8.6)	< 0.001
Acute myocardial injury	58 (0.2)	18 (0.1)	40 (0.3)	0.003
Acute hepatitis	58 (0.2)	20 (0.2)	38 (0.3)	0.014
Acute renal failure	324 (1.3)	76 (0.6)	248 (1.9)	< 0.001
Shock	116 (0.5)	51 (0.4)	65 (0.5)	0.152

*NTD* New Taiwan Dollars, *EUR* Euro, *IQR* interquartile range

^a^Comparison between females and males

^b^Spring: March, April, and May; summer: June, July, and August; autumn: September, October, and November; winter: December, January, and February

^c^Median (IQR) = 16,500 NTD (1,007 NTD − 21,000 NTD)

^d^According to the exchange rate between NTD and EUR in 2012: 1,007 NTD = 31.2 EUR; 16,500 NTD = 511.2 EUR; 21,000 NTD = 649.0 EUR

**Table 2:** Treatments, mortality, and morbidities in patients of carbon monoxide poisoning in Taiwan between 1999 and 2012

Variable	Total N (%)	Female N (%)	Male N (%)	*p*-value^a^
Cases	25,912 (100.0)	13,111 (50.6)	12,801 (49.4)	
Treatment				
Hyperbaric oxygen therapy				0.450
Yes	6,279 (24.2)	3,151 (24.0)	3,128 (24.4)	
No	19,633 (75.8)	9,960 (76.0)	9,673 (75.6)	
Cardiopulmonary resuscitation	548 (2.1)	243 (1.9)	305 (2.4)	0.003
Treatment place				
ED	18,986 (73.3)	9,789 (74.7)	9,197 (71.9)	< 0.001
Transfer to hospitalization care	4,062 (21.4)	1,901 (19.4)	2,161 (23.5)	< 0.001
Hospitalization care only	6,926 (26.7)	3,322 (25.3)	3,604 (28.2)	
Length of stay (day)				
ED to hospitalization care, median (IQR)^b^	3 (2–6)	2 (1–3)	2 (1–4)	< 0.001
Hospitalization stay, median (IQR)^c^	3 (1–6)	3 (1–5)	3 (2–6)	< 0.001
Mortality				
Immediate death	104 (0.4)	46 (0.4)	58 (0.5)	0.193
1 month	407 (1.6)	168 (1.3)	239 (1.9)	< 0.001
3 months	1,285 (5.0)	518 (4.0)	767 (6.0)	< 0.001
6 months	1,554 (6.0)	602 (4.6)	952 (7.4)	< 0.001
1 year	1,838 (7.1)	690 (5.3)	1,140 (8.9)	< 0.001
2 years	2,231 (8.6)	857 (6.5)	1,374 (10.7)	< 0.001
5 years	2,900 (11.2)	1,097 (8.4)	1,803 (14.1)	< 0.001
Morbidity				
Neurological sequelae	2,349 (9.1)	1,090 (8.3)	1,259 (9.8)	< 0.001
2 weeks	602 (2.3)	242 (1.9)	360 (2.8)	< 0.001
6 weeks	923 (3.6)	355 (2.7)	568 (4.4)	< 0.001
6 months	1,209 (4.7)	482 (3.7)	727 (5.7)	< 0.001
12 months	1,369 (6.2)	570 (4.4)	799 (6.2)	< 0.001
Chronic respiratory failure	1,282 (5.0)	505 (3.9)	777 (6.1)	< 0.001
Long-term care	16 (0.1)	5 (0.04)	11 (0.1)	0.122

*ED* emergency department, *IQR* interquartile range

^a^Comparison between females and males

^b^Data for the patients who were transferred to hospitalization care from emergency department (*N* = 4,062)

^c^Data for the patients who received hospitalization care only (*N* = 6,926)

In comparison with female patients, male patients were older (36.8 vs. 35.2 years, *p* < 0.001). Male patients also had a higher proportion attributable to suicide attempts (22.9% vs. 17.8%, *p* < 0.001) and higher incidence rates of acute respiratory failure, acute myocardial injury, and acute hepatitis. Whereas male patients were more likely to require CPR (2.4% vs. 1.9%, *p* = 0.003), they were not more likely to receive HBOT. Male patients had higher 1-month, 3-month, 6-month, 1-year, 2-years, and 5-years mortality rates and a higher incidence of neurological sequelae. While male patients were more likely to develop chronic respiratory failure (6.1% vs. 3.9%, *p* < 0.001), the difference in the proportion of needing long-term care did not reach statistical significance (0.1% vs. 0.04%, *p* = 0.122), most likely due to the small case numbers (16 in total). Table 3 showed the incidence rate of COP in age subgroups and Table 4 showed the incidence rate of COP in four residential regions in Taiwan.

**Table 3:** The incidence rate of carbon monoxide poisoning in age subgroups

Variable	Carbon monoxide poisoning (number of new cases)						Incidence (per 10,000)					
	< 20	20–35	35–50	50–65	65–80	> 80	< 20	20–35	35–50	50–65	65–80	> 80
Year												
1999	107	104	85	28	20	10	0.2	0.2	0.2	0.1	0.1	0.4
2000	100	147	107	44	20	7	0.2	0.3	0.2	0.2	0.1	0.2
2001	132	239	192	50	31	11	0.2	0.4	0.4	0.2	0.2	0.3
2002	159	424	353	109	50	13	0.3	0.7	0.6	0.4	0.3	0.4
2003	204	564	428	106	55	21	0.4	1.0	0.8	0.3	0.3	0.6
2004	245	676	557	170	55	23	0.4	1.2	1.0	0.5	0.3	0.6
2005	392	1,111	843	271	107	35	0.7	2.0	1.5	0.8	0.6	0.8
2006	238	885	811	288	80	27	0.4	1.6	1.5	0.8	0.4	0.6
2007	220	809	710	303	86	31	0.4	1.5	1.3	0.8	0.5	0.6
2008	330	948	811	335	103	52	0.6	1.7	1.5	0.8	0.6	1.0
2009	301	955	777	336	103	35	0.6	1.7	1.4	0.8	0.6	0.6
2010	354	982	801	420	123	36	0.7	1.8	1.4	1.0	0.7	0.6
2011	359	866	756	405	123	59	0.7	1.6	1.4	0.9	0.7	0.9
2012	321	947	784	426	125	46	0.7	1.8	1.4	0.9	0.6	0.7

**Table 4:** The incidence rate of carbon monoxide poisoning in four residential regions in Taiwan

Variable	Carbon monoxide poisoning (number of new cases)				Incidence (per 10,000)			
	North	Center	South	East	North	Center	South	East
Year								
1999	203	56	90	5	0.2	0.1	0.1	0.1
2000	232	78	108	7	0.2	0.2	0.2	0.1
2001	412	99	136	8	0.4	0.2	0.2	0.1
2002	730	146	215	17	0.7	0.3	0.3	0.3
2003	823	232	262	61	0.8	0.5	0.4	1.0
2004	1,117	251	320	38	1.1	0.6	0.4	0.6
2005	1,625	414	662	58	1.5	0.9	0.9	1.0
2006	1,288	381	616	44	1.2	0.9	0.9	0.8
2007	1,298	343	475	43	1.2	0.8	0.7	0.7
2008	1,677	334	520	48	1.5	0.7	0.7	0.8
2009	1,550	445	469	43	1.4	1.0	0.7	0.7
2010	1,695	460	513	48	1.5	1.0	0.7	0.8
2011	1,712	379	443	34	1.6	0.8	0.6	0.6
2012	1,592	505	517	35	1.4	1.1	0.7	0.6

**Fig. 2:**
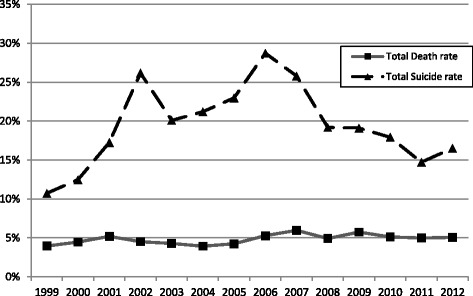
Trends of suicide and 3-month mortality rates in COP patients in Taiwan between 1999 and 2012. Total death rate: number of death/number of all COP patients. Total suicide rate: number of suicide/number of all COP patients

**Fig. 3:**
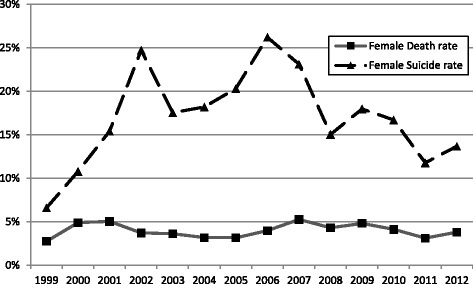
Trends of suicide and 3-month mortality rates in female COP patients in Taiwan between 1999 and 2012. Female death rate: number of death in the female patients with COP/number of all female patients with COP. Total suicide rate: number of suicide in the female patients with COP/number of all female patients with COP

**Fig. 4:**
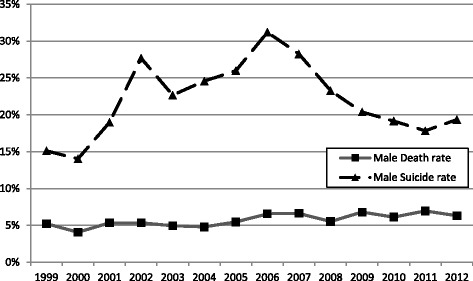
Trends of suicide and 3-month mortality rates in male COP patients in Taiwan between 1999 and 2012. Male death rate: number of death in the male patients with COP/number of all male patients with COP. Male suicide rate: number of suicide in the male patients with COP/number of all male patients with COP

The 3-month mortality rate did not have significant changes between 1999 and 2012, which was around 5% over the years (Fig. 2). The proportion of patients resulted from suicide attempts and increased from 1999, reached the peak in 2007, and then decreased gradually. The trends were similar between the two sexes (Figs. 3 and 4).

## Discussion

This nationwide study showed a whole picture of COP in Taiwan between 1999 and 2012. Most of the patients were less than 50 years old, and the mean age was 36.0 years, which is compatible with a report from a poison center (33 years) [26] and a smaller nationwide study (36 years) [6]. Most COP patients were from the middle income group, and nearly one-third had mental disorders, also similar to the findings reported by the poison center [26].

In Taiwan, COP is more common in winter because of the fact that people are more likely to close windows while using water heaters, which causes inadequate ventilation that leads to production and accumulation of CO in the house [27]. In this study, about 60% of the patients lived in North Taiwan and only 1.9% patients lived in East Taiwan. This is compatible with the fact that the population in North Taiwan is much larger than that in East Taiwan. Our study showed that about 80% patients were less than 50 years old and only 5.8% patients were more than 65 years old which is compatible with another study in a poison center in Taiwan [26]. One of the possible explanations is that young and middle age people are more vulnerable to commit suicide by charcoal burning than the elderly [18, 19]. Studies have shown that the unintentional death rate from COP in Taiwan increased from 1.6 to 3.5 per10^6^ person-years between 1993 and 2003, and most of the cases were caused by to the use of water heaters [28, 29]. Therefore, the Taiwan National Fire Agency has been educating the citizens to prevent COP caused by domestic use of water heaters [29].

We found that mental disorder was the most common underlying comorbidity of COP patients. Some of them may be attributable to suicide attempts by patients with illness like depression. Except mental disorder, male patients had higher prevalence of alcohol abuse than female patients, which was compatible with the finding in general population in Taiwan [30]. Suicide attempt by charcoal burning is an important etiology of COP in Taiwan, and we found that about one of five COP cases was a suicidal attempt. A hospital-based study observed a proportion up to 49.4% [26]. Suicide attempt by charcoal burning in this study was highest in the spring, but the proportion was quite close to the expected value (25%), and therefore we can infer that suicide is independent of season. Since the first case was reported vividly by the media as a painless, nonviolent way to end one’s life in Hong Kong, charcoal burning suicide has increased in the local area and spread to other communities, as well [19]. Because Taiwan is close to Hong Kong and shares a similar background of culture, the incidence of charcoal burning suicide also increased rapidly in Taiwan [19]. A study reported that charcoal burning suicide led to an increase of more than 20% in the overall suicide rates in Hong Kong and urban Taiwan between 1997 and 2002 [19]. We observed a similar pattern of increasing between 1999 and 2002. In addition to Hong Kong and Taiwan, other Asian countries, including Macau, Japan [31], and Korea [32] had similar findings. In 2005, the Taiwan Department of Health assigned the Taiwanese Society of Suicidology the task of setting up the Taiwan Suicide Prevention Center [33], with the mission of preventing suicide and facilitating an efficient care delivery networks nationwide [33]. One of its high-priority tasks is to prevent charcoal burning suicide [33]. Our study results showed that the proportion of suicide attempts in COP patients decreased gradually after 2007, which may be attributed to the effort of the Taiwan Suicide Prevention Center. There was a low percentage of patients with COP had concomitant burn in this study. While there is no study about this issue in Taiwan in the literature indexed by PubMed, a study in the United States reported the only 1.2% patients with burn had concomitant COP [34]. This suggests that burns may contribute only a small portion of COP in some countries. In addition to suicide, the remaining are mostly due to accidental poisoning, including those from fires with or without burns.

HBOT was used in 24.2% COP patients, which was close to the finding in a hospital-based study (18.8%) [26]. Using HBOT instead of using NRM to treat COP is still a controversy nowadays. A recent Cochrane review reported that existing randomized trials do not establish whether the administration of HBOT to patients with COP reduces the incidence of neurological sequelae. The authors suggested that additional research such as a multicenter randomized controlled trial is needed to better define the role, if any, of HBOT in the treatment of COP patients [12].

Neurological sequelae are of great concern in COP, and our study showed that 9.1% of COP patients had neurological sequelae. We found that about 5% of COP patients suffered from chronic respiratory failure that required respiratory treatment and that 0.1% of COP patients were eventually admitted to a long-term care facility. Due to the lack of data regarding this issue in the literature, further studies on the economic and social burden of chronic respiratory failure and long-term care are needed.

Because our study included almost all the COP patients during the study period in Taiwan, we believe that it can add to the current knowledge of COP. Nonetheless, there are some limitations. First, we used surrogates to estimate some diagnoses or conditions because the NPD lacked some detailed information, which may underestimate the real numbers of the patients. Second, the NPD lacked details of sociodemographic characteristics, such as education level, stress level, body mass index, and alcohol drinking habits; results of clinical examinations; laboratory data such as COHb; and causes of death. Therefore, we could not address these issues in this study. Third, the result showed that about 0.1% COP patients needed long-term care after following up; however, it did not indicate that the major cause for long-term care in all these patients was COP itself. Fourth, neurological sequelae were defined as new-onset neurological or psychiatric diseases related to COP which might not be sufficient to provide a causal relationship. This is the congenital limitation of the database; however, we believe the definition adapted in this study is the best method. Fifth, we could not clarify the percentage of immediate death caused fire because there was no data about fire in NPD. Furthermore, although this is a nationwide study, the results may not be applicable to other nations because of the differences in race and culture. Further studies are warranted to clarify these issues above.

## Conclusions

This nationwide descriptive analysis showed a whole picture of the demographic characteristics and short-term and long-term morbidity and mortality of COP patients in Taiwan, which could add to the current knowledge of COP. Some important findings, including the characteristics of the patients, higher percentages of mental disorders in female patients and suicide attempt in male patients, seasonal changes, and trends in mortality and morbidity (suicide) rates may help us develop strategies for the prevention and treatment of COP.

## Additional file

Additional file 1: Table S1. Variables and corresponding ICD-9-CM, management code or drug code, and case classification code. (DOCX 14 kb)

## References

[1] United States Environmental Protection Agency. An Introduction to Indoor Air Quality (IAQ). Carbon Monoxide (CO). http://www.epa.gov/iaq/co.html. Accessed 22 March 2016.

[2] CDC (Centers for Disease Control and Prevention) (2005). Unintentional, non-fire-related, carbon monoxide exposures--United States, 2001-2003. MMWR Morb Mortal Wkly Rep.

[3] Prockop LD, Chichkova RI (2007). Carbon monoxide intoxication: an updated review. J Neurol Sci.

[4] Ernst A, Zibrak JD (1998). Carbon monoxide poisoning. N Engl J Med.

[5] Weaver LK (2009). Clinical practice. Carbon monoxide poisoning. N Engl J Med.

[6] Huang CC, Chung MH, Weng SF, Chien CC, Lin SJ, Lin HJ, Guo HR, Su SB, Hsu CC, Juan CW (2014). Long-term prognosis of patients with carbon monoxide poisoning: a nationwide cohort study. PLoS One.

[7] Zou JF, Guo Q, Shao H, Li B, Du Y, Liu M, Liu F, Dai L, Lin HJ, Su SB, Guo HR, Huang CC (2015). Lack of pupil reflex and loss of consciousness predict 30-day neurological sequelae in patients with carbon monoxide poisoning. PLoS One.

[8] Zou JF, Guo Q, Shao H, Li B, Du Y, Liu M, Liu F, Dai L, Chung MH, Lin HJ, Guo HR, Yang TM, Huang CC, Hsu CC (2014). A positive Babinski reflex predicts delayed neuropsychiatric sequelae in Chinese patients with carbon monoxide poisoning. Biomed Res Int.

[9] Buckley NA, Isbister GK, Stokes B, Juurlink DN (2005). Hyperbaric oxygen for carbon monoxide poisoning: a systematic review and critical analysis of the evidence. Toxicol Rev.

[10] Juurlink DN, Buckley NA, Stanbrook MB, Isbister GK, Bennett M, McGuigan MA (2005). Hyperbaric oxygen for carbon monoxide poisoning. Cochrane Database Syst Rev.

[11] Henry JA (2005). Hyperbaric therapy for carbon monoxide poisoning: to treat or not to treat, that is the question. Toxicol Rev.

[12] Buckley NA, Juurlink DN, Isbister G, Bennett MH, Lavonas EJ (2011). Hyperbaric oxygen for carbon monoxide poisoning. Cochrane Database Syst Rev.

[13] Weaver LK, Hopkins RO, Chan KJ, Churchill S, Elliott CG, Clemmer TP, Orme JF, Thomas FO, Morris AH (2002). Hyperbaric oxygen for acute carbon monoxide poisoning. N Engl J Med.

[14] Huang CC, Ho CH, Chen YC, Lin HJ, Hsu CC, Wang JJ, Su SB, Guo HR. Hyperbaric oxygen therapy is associated with lower short- and long-term mortality in patients with carbon monoxide poisoning. Chest. 2017 Apr 17. pii: S0012–3692(17)30723–7. doi: 10.1016/j.chest.2017.03.049. [Epub ahead of print]. 10.1016/j.chest.2017.03.049 28427969

[15] Sircar K, Clower J, Shin MK, Bailey C, King M, Yip F (2015). Carbon monoxide poisoning deaths in the United States, 1999 to 2012. Am J Emerg Med.

[16] Centers for Disease Control and Prevention. 2016. Carbon Monoxide poisoning fact sheet. http://www.cdc.gov/co/pdfs/faqs.pdf. Accessed 8 Mar 2016.

[17] Braubach M, Algoet A, Beaton M, Lauriou S, Héroux ME, Krzyzanowski M (2013). Mortality associated with exposure to carbon monoxide in WHO European Member States. Indoor Air.

[18] Pan YJ, Liao SC, Lee MB (2010). Suicide by charcoal burning in Taiwan, 1995-2006. J Affect Disord.

[19] Liu KY, Beautrais A, Caine E, Chan K, Chao A, Conwell Y, Law C, Lee D, Li P, Yip P (2007). Charcoal burning suicides in Hong Kong and urban Taiwan: an illustration of the impact of a novel suicide method on overall regional rates. J Epidemiol Community Health.

[20] National Health Insurance Administration, Ministry of Health and Welfare, Taiwan, R.O.C. (2014). National Health Insurance Annual Report 2014–2015.

[21] Statistics Canada. Range and quartiles. http://www.statcan.gc.ca/edu/power-pouvoir/ch12/5214890-eng.htm#a1. Accessed 13 May 2017.

[22] Collins TC, Petersen NJ, Menke TJ, Souchek J, Foster W, Ashton CM (2003). Short-term, intermediate-term, and long-term mortality in patients hospitalized for stroke. J Clin Epidemiol.

[23] Mumma BE, Diercks DB, Holmes JF (2014). Availability and utilization of cardiac resuscitation centers. West J Emerg Med.

[24] National Health Insurance Administration, Ministry of Health and Welfare. Classification of diseases. http://www.nhi.gov.tw/webdata/webdata.aspx?menu=18&menu_id=703&webdata_id=1008. Accessed 27 Mar 2017.

[25] National Health Insurance Research Database. Coding book. http://nhird.nhri.org.tw/date_02.html. Accessed 27 Mar 2017.

[26] Ku CH, Hung HM, Leong WC, Chen HH, Lin JL, Huang WH, Yang HY, Weng CH, Lin CM, Lee SH, Wang IK, Liang CC, Chang CT, Lin WR, Yen TH (2015). Outcome of patients with carbon monoxide poisoning at a far-east poison center. PLoS One.

[27] Heckerling PS (1987). Occult carbon monoxide poisoning: a cause of winter headache. Am J Emerg Med.

[28] Shie HG, Li CY (2007). Population-based case-control study of risk factors for unintentional mortality from carbon monoxide poisoning in Taiwan. Inhal Toxicol.

[29] National Fire Agency, Ministry of the Interior. http://www.nfa.gov.tw/main/Unit.aspx?ID=&MenuID=500&ListID=315. Accessed 3 Aug 2016.

[30] Taiwan Ministry of Health and Welfare. Analysis of sex difference in disease. www.mohw.gov.tw/cht/DOS/DisplayStatisticFile.aspx?d=12117&s=1. Accessed 30 Mar 2017.

[31] Yip PS, Lee DT (2007). Charcoal-burning suicides and strategies for prevention. Crisis.

[32] Huh GY, Jo GR, Kim KH, Ahn YW, Lee SY (2009). Imitative suicide by burning charcoal in the southeastern region of Korea: the influence of mass media reporting. Leg Med (Tokyo).

[33] Taiwan suicide prevention center. http://tspc.tw/tspc/portal/center/index.jsp?sno=93. Accessed 3 Aug 2016.

[34] Williams J, Lewis RW, Kealey GP (1992). Carbon monoxide poisoning and myocardial ischemia in patients with burns. J Burn Care Rehabil.

