# Supplementary material for: Demographic and clinical characteristics of carbon monoxide poisoning: nationwide data between 1999 and 2012 in Taiwan
Source: Scand J Trauma Resusc Emerg Med. 2017 Jul 14;25:70. doi: 10.1186/s13049-017-0416-7 (PMC5512843; doi:10.1186/s13049-017-0416-7)
Supplement: Additional file 1: Table S1. — Variables and corresponding ICD-9-CM, management code or drug code, and case classification code. (DOCX 14 kb) [file 13049_2017_416_MOESM1_ESM.docx]

**Table S1.** Variables and corresponding ICD-9-CM, management code or drug code, and case classification code.

| Variable | ICD-9-CM | Management code or drug code | Case classification code |
| --- | --- | --- | --- |
| Carbon monoxide poisoning | 986, E868, E952, or E982 | − | − |
| Alcohol abuse | 291, 303, 3050, 3575, 4255, 5353, 5710-5713, or V113 | − | − |
| Mental disorder | 290-319 | − | − |
| Suicide | E950-E959 | 94.0 or 94.1 | − |
| Drug poisoning | 960-989, exclusion of 986 | − | − |
| Burn | 940-949 | − | − |
| Acute respiratory failure | 518.81 or 518.84 | 960, 9601, 9602, 9603, 9604, 9605, 9390, 9391, or 311 | − |
| Acute myocardial injury | 410 | − | − |
| Acute hepatitis | 573.3 | − | − |
| Acute renal failure | 584 | 339.5 | − |
| Shock | 785.5 | Use of dopamine (drug code: A032705221, A032792229, A049624265, AC21978221, AC32704221, B015749221, BC23340265, BC23341265, BC23361265, BC25227221) or norepinephrine (drug code: AC55025219, BC22013219, BC25322219) | − |
| Cardiopulmonary resuscitation | − | 960 or 47029C | − |
| Hyperbaric oxygen therapy | − | 47054C, 9395, 59003B, 59004B, 59003A, or 59004A | − |
| Immediate death | Diagnosis of Carbon monoxide poisoning combined with 427.5, 798 or 799 | − | − |
| Neurological sequelae | 290, 293-298, 300, or 331-352 | − | − |
| Chronic respiratory failure | 518.83 or 518.84 | 312 or 967 | − |
| Long-term care | − | − | 61, 62, 65, 66, or 67 |
